# CDK4 regulates cancer stemness and is a novel therapeutic target for triple-negative breast cancer

**DOI:** 10.1038/srep35383

**Published:** 2016-10-19

**Authors:** Meiou Dai, Chenjing Zhang, Ayad Ali, Xinyuan Hong, Jun Tian, Chieh Lo, Nadège Fils-Aimé, Sergio A. Burgos, Suhad Ali, Jean-Jacques Lebrun

**Affiliations:** 1 Department of Medicine, McGill University Health Center, Cancer Research Program, Montreal, Quebec, H4A 3J1, Canada; 2 Department of Animal Science, McGill University, Sainte-Anne-de-Bellevue, H9X 3V9, Canada

## Abstract

Triple negative breast cancers exhibit very aggressive features and poor patient outcomes. These tumors are enriched in cancer stem cells and exhibit resistance to most treatments and chemotherapy. In this study, we found the cyclin-dependent kinase (CDK4) to act as a cancer stem cell regulator and novel prognostic marker in triple negative breast cancers. We found CDK4 to be highly expressed in these tumors and its expression to correlate with poor overall and relapse free survival outcomes, high tumor grade and poor prognostic features of triple negative breast cancer patients. Moreover, we found that blocking CDK4 expression or kinase activity, using a pharmacological inhibitor prevented breast cancer stem cell self-renewal. Interestingly, suppression of CDK4 expression or kinase activity reversed the basal-B TNBC mesenchymal phenotype to an epithelial- and luminal-like phenotype which correlates with better clinical prognosis. Finally, blocking CDK4 activity efficiently eliminated both normal and chemotherapy-resistant cancer cells in triple negative breast cancers, highlighting CDK4 as a promising novel therapeutic target for these aggressive breast tumors.

Triple negative breast cancers (TNBCs) represent 10–20% of all diagnosed breast cancers and are characterized by their aggressive features leading to poor patient outcome. They are negative for estrogen and progesterone receptors and HER2, as a result they are insensitive to anti-hormonal therapies or therapy targeting HER2 receptors. TNBCs also contain high proportion of breast cancer stem cells (BCSCs) and exhibit resistance to chemotherapy treatments. Thus, there is immense interest in finding and developing novel drugs aiming at efficiently targeting this type of breast cancer.

Over the past decade, a paradigm shift modified our understanding of the process of cancer development, resistance, and cancer recurrence emphasizing the cancer stem cell (CSC) or tumor initiating cell concept. CSCs are capable of driving not only tumor initiation, but also cancer cell metastasis in various human cancer types^1^,^2^. These distinct subpopulations of cancer cells utilize their self-renewal ability to generate and propagate heterogeneous tumors^3^. Remarkably, they display resistance to radiation and chemotherapy based treatments, resulting in increased local and distant tumor relapse, ultimately causing cancer patient death^4^. Distinct CSCs with specific phenotypic markers have been found in many solid tumors such as breast, brain, pancreatic, lung, prostate, and colon carcinomas^5^,^6^,^7^,^8^. Accumulating evidence indicates that eliminating CSCs is a requirement to prevent cancer relapse, drug resistance, and metastasis. Thus, understanding molecular mechanisms governing CSC self-renewal is crucial to the development of new cancer therapies. In breast cancer patients, BCSCs are frequently detected in metastatic pleural effusions or early-disseminated cancer cells within the bone marrow^5^,^9^. Initial studies identified a small subpopulation of BCSCs that express the CD44^+^/CD24^−/low^/Lin^−^ surface markers. These BCSCs are capable of generating new tumors when inoculated in immunodeficient mice, even in numbers as low as 1000 cells, whereas inoculating tens of thousands of cancer cells that do not express these markers failed to initiate tumors. CD44^+^/CD24^−/low^ BCSCs are not the only population with stem-like and tumorigenic properties. Indeed, another subset of BCSCs, enriched in aldehyde dehydrogenase positive (ALDH^+^) cells was recently characterized. These BCSCs are also able to generate mammary tumors in immunodeficient mice^10^. While the CD44^+^/CD24^−^ and ALDH^+^ CSCs exist as two distinct populations, they are also plastic in nature and can switch between the two states^3^,^5^,^10^. While CD44^+^/CD24^−^ BCSCs display a mesenchymal-like feature and are localized at the tumor invasive front, ALDH^+^ BCSCs are epithelial-like and are found at the centre of the tumor. These two BCSC populations are found in all breast cancer molecular subtypes, highlighting their critical role in tumor development and further emphasizing the need for new breast cancer therapies targeting these BCSC populations.

The cell cycle regulator, CDK4 and its binding partner cyclin D1 are the ultimate targets of many oncogenic signals, suggesting a central role for these proteins in cancer development and progression^11^,^12^,^13^. These proteins are often deregulated in human cancers^11^, and a comprehensive analysis of somatic copy-number alterations from thousands of tumor specimens identified the CDK4 gene to be the most frequently amplified in human cancers^14^. Interestingly, CDK4 was also shown to promote normal stem cell expansion and inhibit differentiation of embryonic, hematopoietic, neural and mammary progenitors^15^,^16^,^17^,^18^. As cancer stem cells are key to tumor development and tumor propagation, we hypothesized that CDK4 may play an important role in regulating BCSC stemness, tumor relapse and drug resistance.

In this study, we investigated the role and function of CDK4 in BCSCs-mediated self-renewal and chemotherapy resistance. We found CDK4 to be highly expressed in TNBCs and to correlate with poor overall survival and relapse free survival outcomes as well as with poor prognostic features of TNBC patients. We found that knocking down CDK4 expression in TNBCs specifically reduced the CD44^+^/CD24^−^ BCSC population and inhibited their self-renewal. Furthermore, blocking CDK4 kinase activity also specifically eliminated the BCSC population and prevented their self-renewal. Interestingly, blocking CDK4 expression or activity led to a switch in TNBCs, from a stem-like basal-B phenotype to a more differentiated epithelial- or luminal-like phenotype. Finally, we showed that TNBC resistance to chemotherapy treatment led to an increase and expansion of the two BCSC populations and that blocking CDK4 activity efficiently reduce the numbers of these chemotherapy-resistant BCSCs. Together, these results highlight CDK4 as a potent BCSC self-renewal regulator and as a critical mediator of TNBC resistance to chemotherapy. Interestingly, we also found these effects to be CDK4-specific and CDK6-independent, indicating that specific CDK4 pharmacological inhibitors, may prove efficient drugs for eliminating BCSCs and further preventing tumor recurrence in human TNBCs.

## Results

### CDK4 is highly expressed in triple-negative breast tumors and correlates with poor prognostic clinical features

**Figure 1:**
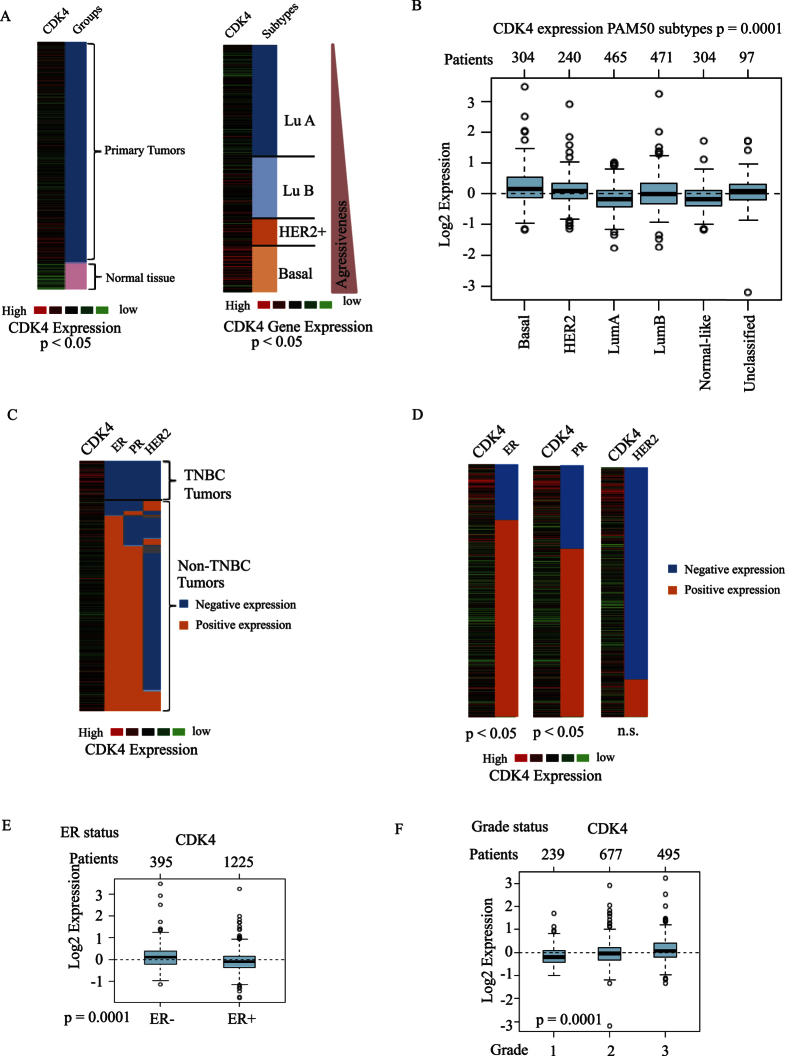
(**A**) Gene set analysis of CDK4 expression in normal tissue and primary breast tumor using TCGA cancer browser (left panel). Gene set analysis of CDK4 expression in 1,881 primary breast tumor which were categorized into different subgroups including basal-like, HER2+, luminal B (Lu B), and luminal A (Lu A) (right panel). (**B**) Box plot of CDK4 gene expression in different subtypes of breast cancer using GOBO gene set analysis. The number of patients from each subtype is indicated above the box plot. (**C**,**D**) CDK4 gene expression in TNBC and non-TNBC patients, divided according to ER, PR and HER expression. (**E**) Box blot of CDK4 expression in primary breast tumors classified according to ER status. (**F**) Box blot of CDK4 expression in primary breast tumors classified into grade 1, grade 2 and grade 3 tumors.

Being the most frequently amplified gene in human cancers, and because of its role in promoting normal stem cell expansion and mammary progenitors, CDK4 may represent a key regulator of BCSC stemness, tumor relapse and drug resistance in breast cancer. The association of CDK4 gene expression with various breast cancer subtypes and clinical prognosis in human patients has not been studied. Thus, we first investigated and compared CDK4 gene expression in various subtypes of human breast cancer, by performing a bioinformatics analysis of CDK4 gene expression using TCGA RNA-seq datasets of 1,215 primary human breast tumors and GOBO microarray datasets of 1,881 primary breast tumors^19^,^20^,^21^. As expected, we found that CDK4 is overexpressed in primary breast tumors compared with normal tissues (Fig. 1A, left panel). Interestingly, our analysis of this large cohort of breast cancer patients using both TCGA RNA-seq and GOBO microarray datasets showed that CDK4 is expressed at the highest level in the most aggressive basal-like subtype, but at the lowest level in the luminal-A subtype normally exhibiting better clinical outcomes (Fig. 1A right panel and 1B). The basal-like subgroup contains most of TNBC patients which have the worst survival outcome compared with other subtypes at 5 years of diagnosis^22^. We found CDK4 gene expression to be highly enhanced in specific triple-negative breast tumors (Fig. 1C). In particular, CDK4 expression significantly correlated with ER and PR negative, but not HER2 status of breast cancers (Fig. 1D,E). Breast tumor grade is also a prognosis indicator of the differentiation stage and growth rate of tumor cells. While tumors of grade 1 and 2 are well or moderately differentiated, respectively, with a slow growth index, grade 3 tumors are poorly differentiated and exhibit fast growth features. In our study, we assessed CDK4 gene expression in relation to the three grades of breast tumors. As shown in Fig. 1F, high CDK4 expression correlates with higher tumor grade. As high grade breast tumors are enriched in BCSCs^23^, these results further suggest that CDK4 may regulate BCSC expansion. Together, our results indicate that CDK4 gene expression is elevated in the aggressive basal-like TNBC subtype and correlates with TNBC poor clinical prognosis features (including ER and PR negative status and high tumor grade) and highlight CDK4 as a potential poor prognostic marker for TNBC patients.

### CDK4 predicts poor survival outcomes and correlates with distant metastatic gene signatures

**Figure 2:**
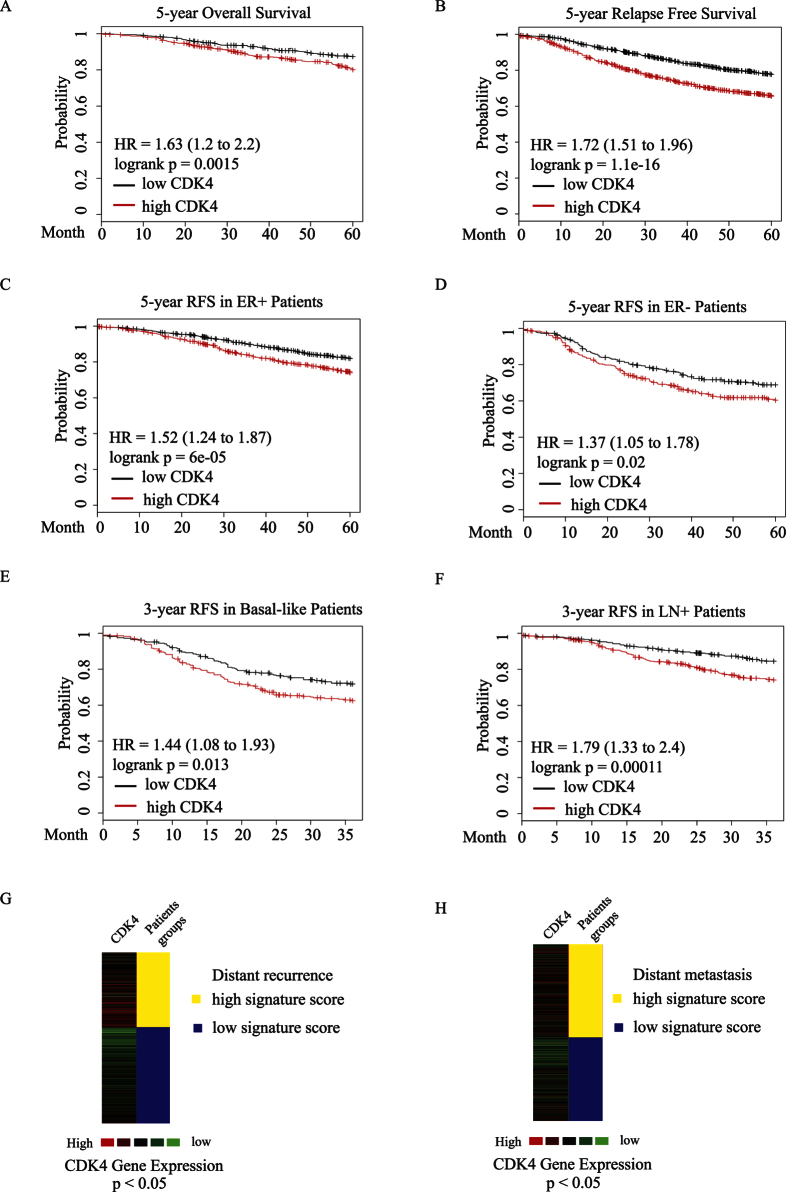
CDK4 expression was assessed by Kaplan-Meier survival analysis for 5-year overall survival outcome in 1,117 breast cancer patients (**A**) as well as 5-year early relapse free survival outcome in 3,554 breast cancer patients (**B**). The survival rates were analyzed using median splits of the patients, based on CDK4 expression levels. High CDK4 expression is represented in red. Hazard ratio (HR). (**C–F**) Correlation analysis between CDK4 expression and relapse free survival outcomes in ER positive, ER negative, basal-like and lymph node positive breast cancer patients. (**G**) CDK4 expression correlates with a high signature score for a set of genes that predicts for distant recurrence in tamoxifen-treated patients with lymph node-negative, ER positive breast cancer. (**H**) CDK4 expression correlates with a high signature score for a set of genes that predicts for poor distant metastasis outcome in the early-stage of TNBC patients.

Due to the absence of well-defined molecular targets, TNBC patients lack effective treatment options and suffer from tumor local relapse and distant metastasis within 5 years following chemotherapy treatment. To assess whether high CDK4 gene expression could predict the clinical survival outcomes of breast cancer patients, we analyzed the overall survival (OS) rate of 1,117 breast cancer patients and early relapse free survival (RFS) rate of 3,554 patients according to the expression levels of CDK4, using Kaplan-Meier plot analysis^24^. As shown in Fig. 2A,B, elevated CDK4 expression significantly correlated with poor OS and RFS outcomes within 5 years. Notably, high CDK4 expression can predict for poor early RFS patients outcome in both ER positive and negative subgroups (Fig. 2C,D). More importantly, CDK4 expression is significantly higher in basal-like subgroup and lymph node positive (LN+) patients with poor RFS outcomes within 3 years, suggesting that enhanced CDK4 expression could be an early indicator for poor RFS outcomes in basal-like and LN+ patients (Fig. 2E,F). We then assessed the relationship between CDK4 and different sets of gene signatures that predicts distant metastatic outcomes. As shown in Fig. 2G, CDK4 expression correlated with a high signature score for a set of genes that predicts a high rate of distant recurrence in tamoxifen-treated patients with lymph node-negative, ER positive breast cancer. In the early-stage of TNBC patients, CDK4 expression also correlated with a high gene signature score for another set of genes that predicts for poor distant metastasis outcome of these TNBC patients (Fig. 2H). Thus, these correlations between CDK4 expression and different distant metastasis/recurrence gene signatures indicate that CDK4 could be used as a useful poor survival outcome indicator for TNBC patients. As previously mentioned, the number of cancer stem cells in breast tumors correlates with clinical aggressiveness and poor relapse free survival outcome in breast cancer patients^23^. Thus, together with our findings linking CDK4 expression and poor clinical outcomes in TNBC patients, our data also suggest that elevated CDK4 expression may play a role in the generation and expansion of cancer stem-like cells or tumor-initiating cells in basal-like TNBC and further lead to TNBC progression.

### Suppression of CDK4 activity or expression specifically reduces the CD44^+^/CD24^−^ BCSC subpopulation of TNBC cells

**Figure 3:**
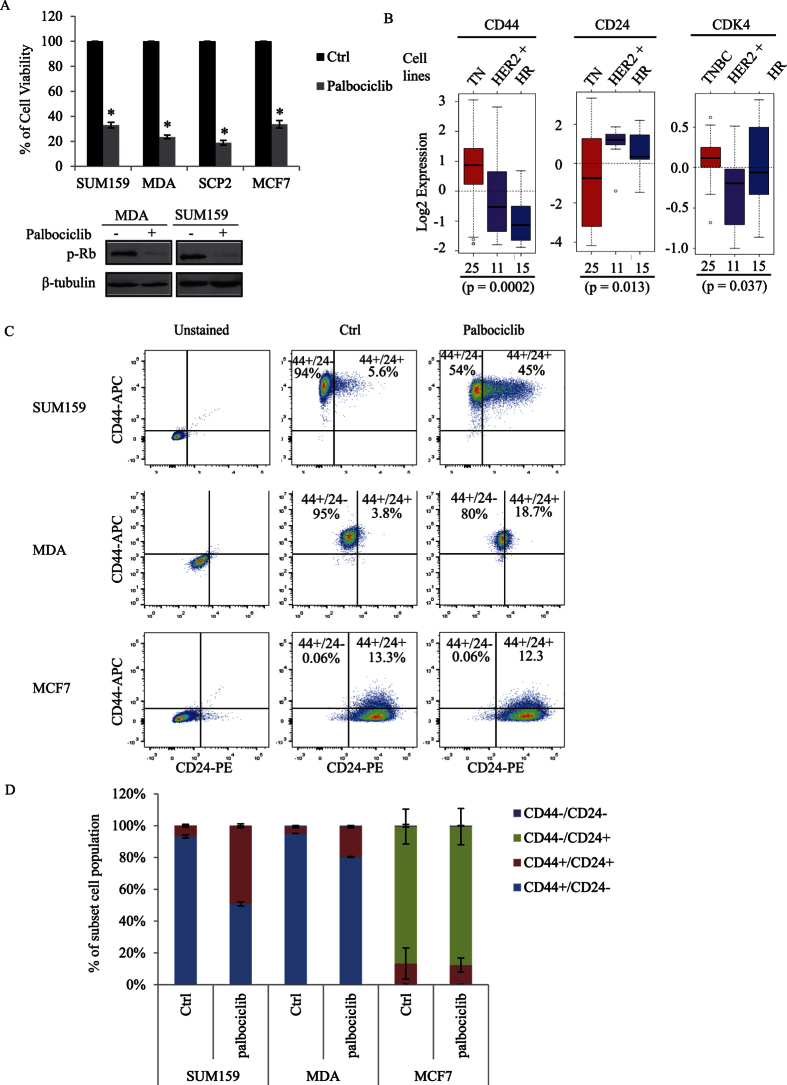
(**A**) SUM159, MDA, SCP2, and MCF7 cells are treated with 100 nM of the CDK4 inhibitor palbociclib for 48 hours. Total cell number was calculated and quantified (upper panel). Total cell lysates were then subjected to immunoblotting using p-RB and β-tubulin antibodies (bottom panel). (**B**) Gene expression of CD44, CD24, or CDK4 was analyzed in different breast cancer subtypes, including triple-negative (TN), HER2 positive (HER2+), and hormone receptor positive (HR) in 51 breast cancer cell lines, using GOBO gene set analysis. (**C**,**D**) SUM159, MDA, and MCF7 cells were treated with or without palbociclib for 2 days, labeled with an anti-CD44 conjugated to APC antibody and with an anti-CD24 conjugated to PE antibody and analyzed by flow cytometry. Percentages of the different CD44^+/−^/CD24^+/−^ subpopulations were graphed and quantified using flowJ.

To investigate the role of CDK4 in cancer stem cell expansion, and to further examine whether CDK4 could be used as a potential drug target for TNBC patients, we used the pharmacological CDK4 inhibitor, palbociclib. Based on its cell cycle regulatory activity, this inhibitor was recently approved by the FDA for treatment in hormone-receptor positive (ER+/PR+) metastatic breast cancer patients^25^. Although this inhibitor is currently used for hormone receptor positive patients, we assessed its effect in TNBCs. We found palbociclib to significantly reduce the cell proliferation rate of several aggressive basal-like TNBC cell lines (SUM159, MDA-MB231, and SCP2). This inhibitory effect is comparable to what is observed in the ER+/PR+ luminal MCF7 cell line, treated with the palbociclib (Fig. 3A). Efficacy of the CDK4 kinase inhibitor, palbociclib was ensured by analyzing the Ser780-phosphorylation levels of retinoblastoma protein (Rb), a known substrate for the CDK4 kinase activity (Fig. 3A)^26^.

**Figure 4:**
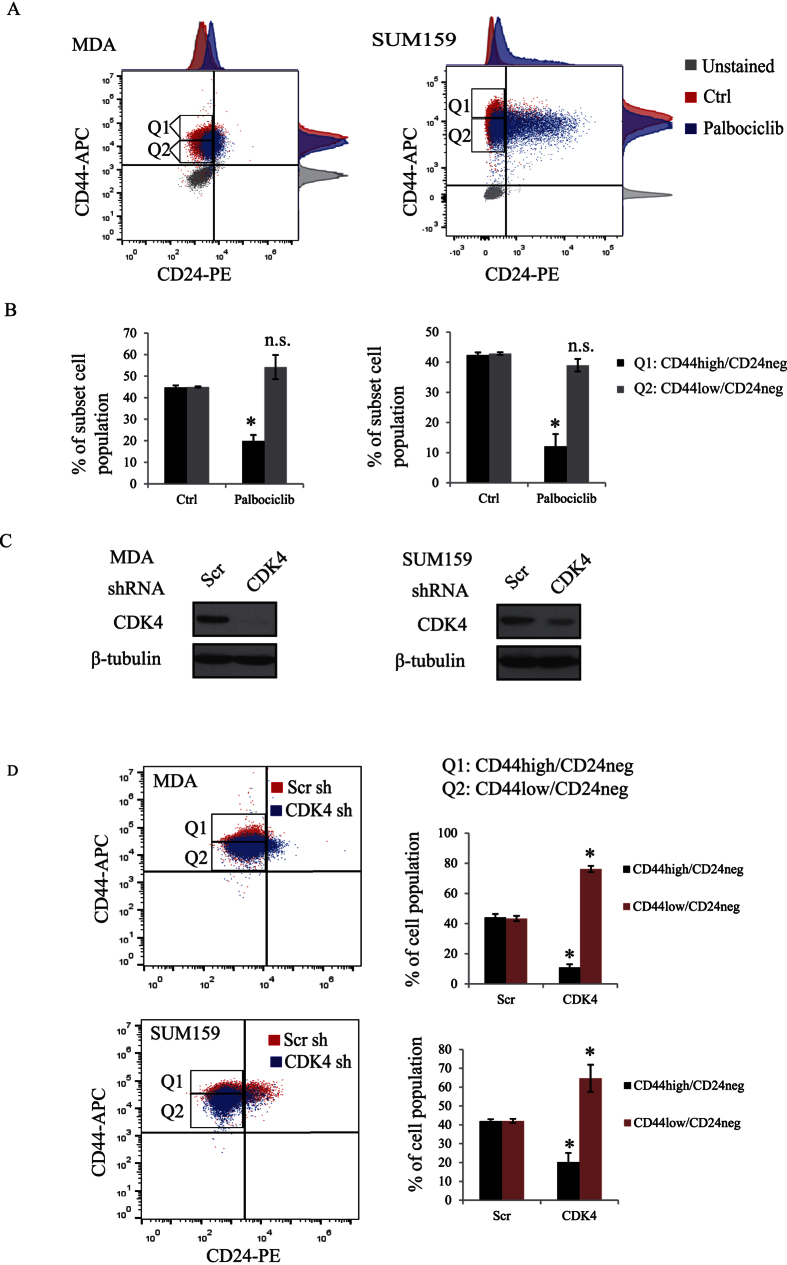
(**A**,**B**) SUM159 and MDA cells were treated with or without palbociclib for 2 days and labeled with anti-CD44-APC and anti-CD24-PE antibodies. Cells were then analyzed by flow cytometry and the percentages of different CD44^high/low^/CD24^neg^ subpopulations were graphed and quantified using flowJ. (**C**) SUM159 and MDA cells were infected with lentiviruses expressing scrambled (scr) or CDK4 shRNAs. Cell lysates were then subjected to immunoblotting using CDK4 and β-tubulin antibodies. (**D**) SUM159 and MDA cells infected with scr or CDK4 shRNA overexpressing lentiviruses were labeled with CD44-APC and CD24-PE antibodies and CD44^high/low^/CD24^neg^ populations were analyzed by flow cytometry.

We next assessed the level of BCSCs in the different breast cancer subtypes by examining the expression levels of the cancer stem cell surface markers CD44 and CD24. As shown in Fig. 3B, using 51 different breast cancer cell lines, we found that TNBC cell lines exhibit higher expression of the cancer stem cell surface marker CD44 and lower expression of the non-CSC CD24 compared with other subtypes. In addition, CDK4 is also highly expressed in these TNBC cell lines (Fig. 3B), suggesting that CDK4 may maintain the CD44^+^/CD24^−^ BCSC population. To further investigate this, TNBC cells (SUM159 and MDA-MB231 (referred as MDA) and luminal cells (MCF7) were treated or not with the CDK4 kinase inhibitor, palbociclib and the percentage levels of the different CD44^+/−^/CD24^+/−^ sub-populations were analysed using flow cytometry. As shown in Fig. 3C,D, TNBC cells contain a high proportion (up to 90%) of the CD44^+^/CD24^−^ BCSC population, compared to a barely detectable level (0.06%) in MCF7 luminal cells. Interestingly, palbociclib treatment of TNBC SUM159 cells led to a significant decrease of the CD44^+^/CD24^−^ BCSC subset (from 93% to 51%) with a concomitant increase of the non-stem cell population, CD44^+^/CD24^+^ (from 6.7% to 49%). A significant decrease of the CD44^+^/CD24^−^ BCSC population (from 95% to 80%) was also observed in MDA cells treated with palbociclib (Fig. 3C,D). In contrast, the CDK4 inhibitor showed no effect in luminal MCF7 cells, which mostly contained non-BCSC populations (CD44^+^/CD24^+^ and CD44^−^/CD24^+^). Since CD44 was identified as a functional CSC marker in colon cancer^27^ and because we found its expression to correlate with CDK4 expression in TNBC cell lines (Fig. 3A), we next examined the percentage levels of CD44^high^/CD24^neg^ and CD44^low^/CD24^neg^ subpopulation in response to palbociclib, using flow cytometry. Interestingly, we found palbociclib to strongly decrease the specific CD44^high^/CD24^neg^ BCSC subpopulation in TNBC cells (Fig. 4A,B). We next examined the effect of knocking down CDK4 gene expression on the CD44^high^/CD24^neg^ subpopulation. The expression of CDK4 was suppressed by infecting MDA or SUM159 cells with lentiviral shRNA targeting CDK4 (Fig. 4C). Consistently, knocking down CDK4 in MDA or SUM159 cells also reduced the percentage of the BCSC CD44^high^/CD24^neg^ subpopulation, suggesting CDK4 specifically maintains BCSC expansion in breast tumors (Fig. 4D). All together, these findings indicate that high expression of CDK4 strongly correlates with cancer stem cell-enriched subtype of breast cancer, specifically the triple-negative group and that the CDK4 kinase activity is required for maintaining the subpopulation of CD44^high^/CD24^neg^ BCSC. These results further suggest that CDK4 play a role in expanding BCSCs and that therapies aiming at targeting CDK4 could efficiently eliminate BCSCs in TNBC patients.

### CDK4 regulates tumorsphere formation and self-renewal of triple-negative BCSCs

**Figure 5:**
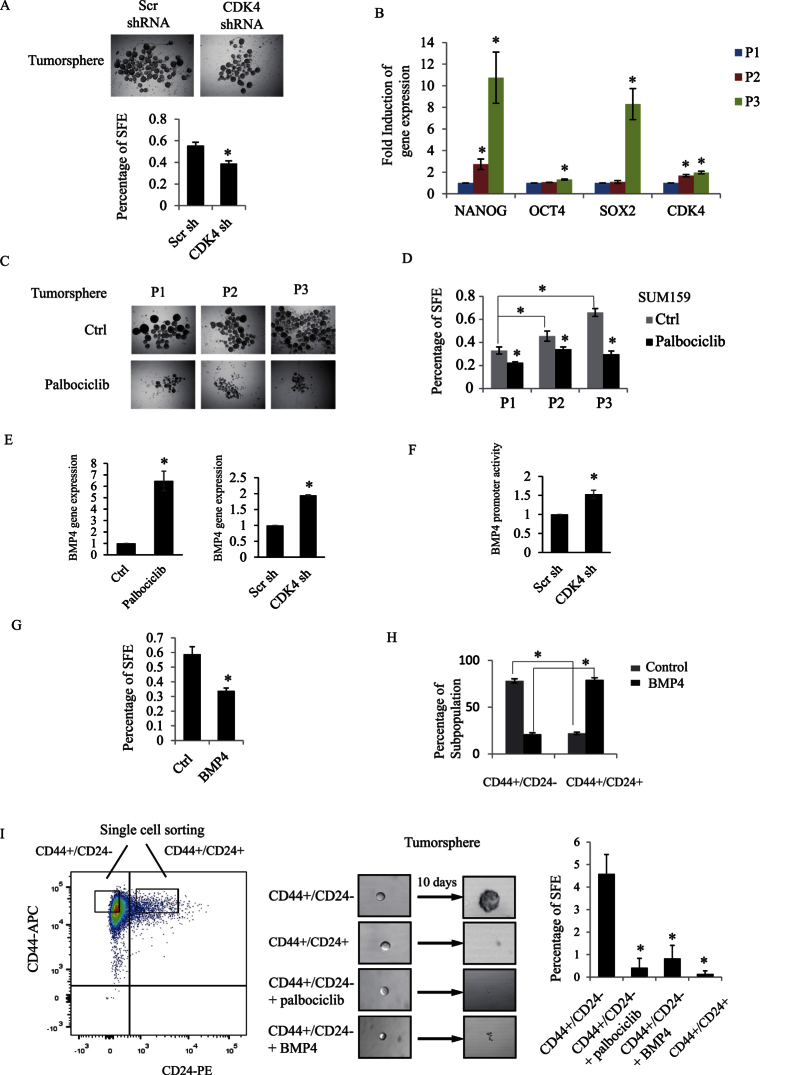
(**A**) SUM159 cells were infected with scrambled or CDK4 shRNA overexpressing lentiviruses. Representative images of infected SUM159 cell-derived tumorspheres in the presence of 20 ng/ml EGF, 20 ng/ml bFGF, and B27 for 7 days. The number of spheres was counted and expressed as percentage of sphere-forming efficiency (SFE). (**B**) Tumorspheres were collected from serial passages (P1, P2 and P3). Total RNA was extracted from these three passages. Gene expression of three stem cell markers (NANOG, OCT4 and SOX2) and of CDK4 were measured by RT-PCR. (**C**) Representative images of tumorspheres (P1, P2 and P3) cultured in the presence or the absence of palbociclib. (**D**) Percentage of SFE from the indicated conditions were quantified and graphed. (**E**) SUM159 cells were treated with palbociclib or infected with scrambled or CDK4 shRNA. Gene expression of BMP4 was measured and graphed. (**F**) Scrambled or CDK4 shRNA infected SUM159 cells were transfected with BMP4 promoter or β-gal construct. Luciferase activity of BMP4 promoter construct was measured and normalized to β-galactosidase. (**G**) SUM159 cells were plated for tumorsphere in the presence or absence of BMP4. Percentage of SFE was measured and graphed. (**H**) SUM159 cells were treated with or without BMP4. The percentage of different CD44/CD24 subpopulations was analyzed and graphed by flowJo. (**I**) Single CD44+/CD24− and CD44+/CD24+ cell were sorted by FACSaria into 96-well low attachment plate for tumorsphere formation and cultured with or without palbociclib or BMP4 for 10 days. Percentage of SFE from the indicated conditions were quantified and graphed.

To understand how CDK4 regulates the CD44^+^/CD24^−^ BCSCs, we assessed their self-renewal capacity, which is a critical feature of BCSC expansion. Previous studies have shown that tumorspheres generated from serum-free medium containing growth factors and low-attachment conditions can be used for assessing BCSC enrichment and BCSCs self-renewal capacity^28^,^29^. While BCSCs are able to form tumorspheres, non-stem-like cells do not generate spheres^30^,^31^. We first examined the effect of knocking down CDK4 in SUM159 on tumorsphere forming efficiency (SFE). As shown in Fig. 5A, knocking down CDK4 expression, by means of shRNA, significantly reduced the percentage of SFE, clearly indicating that CDK4 does regulate BCSC self-renewal capacity.

We then assessed the inhibition of CDK4 kinase activity on BCSC long-term self-renewal abilities, using enriched BCSC populations, generated from primary, secondary and third passages tumorspheres. For this, primary tumorspheres (P1) were dissociated into single cells to allow for the generation of secondary and third passages of tumorspheres (P2 and P3). BCSC enrichment, upon successive passages of the tumorspheres led to increased gene expression of the stem cell self-renewal markers nanog, pou5f1 (coding for Oct4), and sox2 (Fig. 5B). Interestingly, CDK4 gene expression was also simultaneously increased with these stem cell markers (Fig. 5B), indicating that increased CDK4 expression correlates with high expression of stem cell markers and regulates BCSCs self-renewal capacity. To define whether CDK4 controls BCSC long-term self-renewal abilities, P1, P2 and P3 tumorspheres were treated or not with palbociclib and as shown in Fig. 5C,D, while SFE progressively increased with sphere passages, treatment with the CDK4 inhibitor reduced SFE in all serial passaged spheres.

With respect to the underlying mechanisms by which CDK4 regulates stemness in TNBC, we found that the CDK4 effect on BCSC self-renewal is, at least partially, mediated through down-regulation of the bone morphogenetic protein-4 (BMP4), a transforming growth factor-beta family member known to act as an important growth factor for CSC tumorigenic capacity and differentiation factor in CSCs^32^. Indeed, as shown in Fig. 5E, blocking CDK4 activity or knocking down CDK4 gene expression clearly increased BMP4 expression. This effect occurs at the transcriptional level, as blocking CDK4 gene expression also led to an increase in BMP4 gene promoter luciferase activity (Fig. 5F). Furthermore, we found BMP4 to potently inhibit tumorsphere formation and to reduce the CD44^+^/CD24^−^ cancer stem cell population in TNBC cells (Fig. 5G,H). To finally address the role of CDK4 on the self-renewal capacity of the specific subpopulation of CD44^+^/CD24^−^, we sorted CD44^+^/CD24^−^ SUM159 single cells to generate tumorspheres at low density to prevent cell aggregation (10 cells per well in 96-well low attachment plate). We found that sorted CD44^+^/CD24^−^ single cells, cultured under serum-free and non-adherent conditions were able to form tumorspheres while CD44^+^/CD24^+^ non-CSCs could not survive under these conditions (Fig. 5I). Using this assay, we further found that blocking CDK4 kinase activity, using palbociclib prevented the CD44^+^/CD24^−^, BCSC subpopulation to form spheres. Interestingly, stimulating the cells with BMP4 produced the same effect and also blocked CD44^+^/CD24^−^ BCSCs to form spheres (Fig. 5I). Together, these results strongly suggest that blocking CDK4 activity leads to increased expression of the differentiation factor BMP4, resulting in decreased CD44^+^/CD24^−^ BCSC numbers, and further leading to inhibition of breast cancer stemness.

### Suppression of CDK4 expression or kinase activity reverses the basal-B TNBC mesenchymal phenotype to an epithelial- and luminal-like phenotype

**Figure 6:**
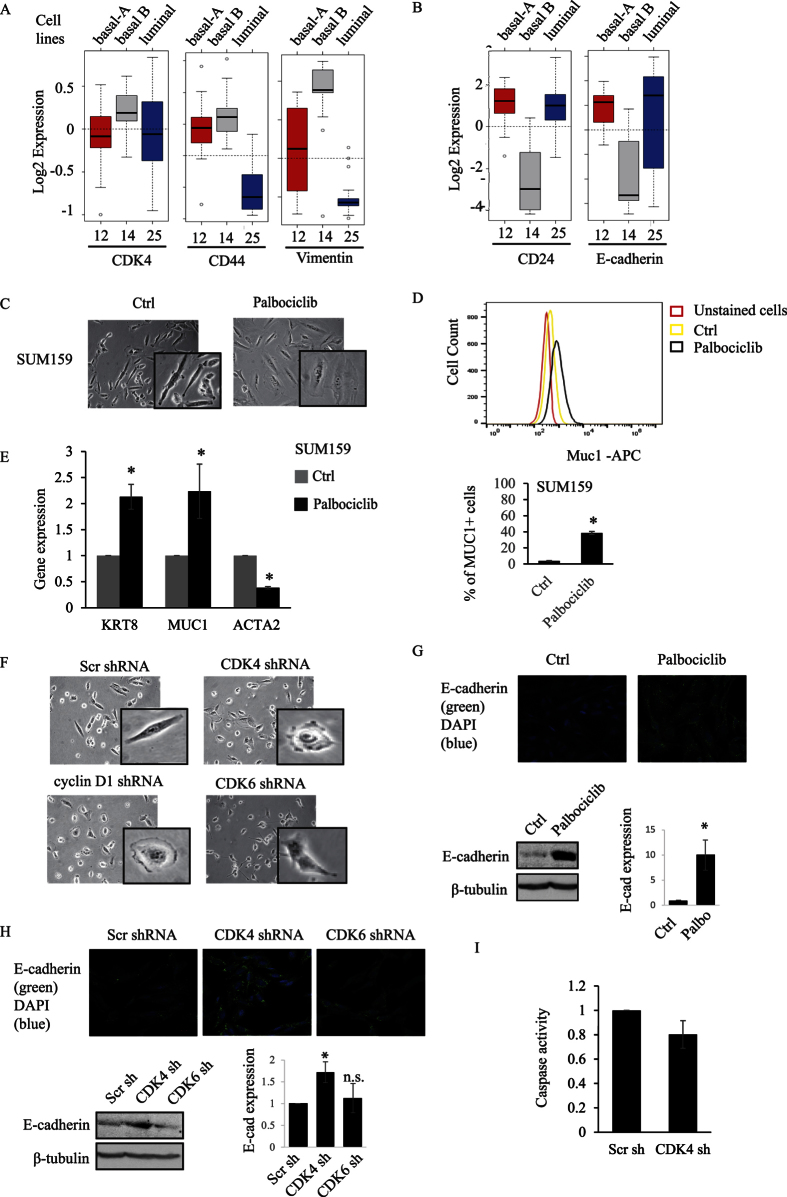
(**A**,**B**) Gene expression of CDK4, CD44, Vimentin, CD24, or E-cadherin was analyzed in 51 breast cancer cell line representing different breast cancer subtypes (basal-A, basal-B and luminal) using GOBO gene set analysis. (**C**) SUM159 cells were treated with or without 100 nM of palbociclib for 2 days and subjected to light microscopy (10× objective). (**D**) Representative histogram of MUC1 population. Percentage of MUC1+ subpopulation was analyzed by flow cytometry in SUM159 cells treated with palbociclib. (**E**) Gene expression of cytokeratin 8 (KRT8), mucin1 (MUC1), and alpha smooth muscle actin (ACTA2) in SUM159 cells were measured using RT-PCR. (**F**) SUM159 cells were infected with scr, cyclin D1, CDK4 or CDK6 shRNA overexpressing lentiviruses and imaged by light microscopy. (**G**) E-Cadherin expression was assessed in SUM159 treated or not with palbociclib, using both immunofluorescence and immunoblot analysis. For Western blots, 3 separate independent experiments were performed and quantified using densitometry. (**H**) SUM159 cells were infected with the scr, CDK4 or CDK6 shRNA lentiviruses. E-Cadherin expression was assessed using both immunofluorescence and immunoblot analysis. For Western blots, 3 separate independent experiments were performed and quantified using densitometry. (**I**) Caspase 3/7 activity of infected SUM159 cells was measured using the Caspase Glo 3/7 Assay.

As CD44^+^/CD24^−^ BCSCs display a mesenchymal-like feature, we examined whether CDK4 expression and activity could correlate with a specific cell phenotype. The expression level of CDK4, CD44, and CD24 was analyzed in 51 breast cancer cell lines containing mesenchymal-like (basal-B) and epithelial-like (basal-A and luminal) phenotypes. As shown in Fig. 6A, basal-B cells express high CD44 and CDK4, along with high levels of the mesenchymal marker vimentin, while basal-A or luminal-like breast cancer cells display higher CD24 level with a high expression level of the epithelial marker E-cadherin (Fig. 6B). Interestingly, we found that inhibition of CDK4 kinase activity using palbociclib led to a change of phenotype in basal-B cells (SUM159, MDA and SCP2) from a mesenchymal-like spindle cell shape into a more spread, epithelial-like feature (Fig. 6C and Supplementary Fig. S1A). Indeed, palbociclib treatment resulted in an increase of a MUC1+ luminal population (Fig. 6D). This change of cell phenotype was also associated with increased expression of the luminal marker cytokeratin KRT8 and Muc1 as well as with a decreased expression of the mesenchymal or basal B marker alpha smooth muscle actin (ACTA2) in SUM159 and MDA (Fig. 6E and Fig. S1B). To further investigate this, we then assessed the effect of knocking down CDK4 gene expression on the basal-B cell phenotype. Depletion of CDK4 in MDA or SUM159 cells consistently resulted in change from mesenchymal-like to luminal-like or epithelial cell phenotype (Fig. 6F and Fig. S1C). Suppression of the CDK4 binding partner and activator cyclin D1 also resulted in a switch towards an epithelial-like cell shape (Fig. 6F). Consistent with this epithelial phenotype, we found that E-cadherin protein expression is restored in mesenchymal SUM159 treated with the CDK4/6 kinase inhibitor palbociclib, as assessed by immunofluorescence and immunoblotting assays (Fig. 6G). These results were further confirmed using a specific CDK4 shRNA to block CDK4 gene expression, also resulting in restoring E-cadherin protein expression, as measured by immunofluorescence and immunoblotting assays (Fig. 6H). Interestingly, these effects appear CDK4 specific as blocking CDK6 gene expression did not change the cell phenotype or E-cadherin expression (Fig. 6F,H). The effects of suppressing CDK4 on MET and phenotypic cell shape changes are not due to cells undergoing apoptosis, as depletion of CDK4 in TNBC cells did not result in an increase in caspase activity (Fig. 6I).

Having found that CDK4 but not CDK6 could regulate MET and the phenotype of the cells, we next investigated the potential role of CDK6 in regulating cancer stemness in TNBC and its potential association with tumor progression and outcomes. Interestingly, as shown in Fig. S2A, knocking down CDK6 expression did not affect tumorsphere formation, suggesting that CDK6 is not required for the self-renewal of BCSCs. Figure S2B indicates the efficiency of the CDK6 shRNA knockdown in these cells. This is in sharp contrast to what observed on the tumorsphere formation when knocking down CDK4 (Fig. 5A). Although CDK6 expression was elevated in basal-like tumor compared with other subtypes of tumors, CDK6 expression was higher in normal tissues compared with primary tumors (Fig. S2C). Moreover, CDK6 expression showed no significant correlation with early overall survival and relapse free survival outcomes of breast cancer patients, including the basal-like breast cancer subgroup (Fig. S2D). This is also opposed to results obtained when knocking down CDK4 (Figs 1 and 2).

All together, these results indicate that blocking CDK4 expression or kinase activity in TNBC cells, not only prevent their self-renewal ability and BCSC expansion but also alters their stem-like mesenchymal phenotype to a more differentiated luminal or epithelial cell type. Our data also rule out CDK6 in the maintenance of breast cancer stemness and indicate that CDK4 is the predominant regulator of cancer stemness, thereby representing a unique and specific prognostic marker for basal-like TNBC patients.

### CDK4 promotes self-renewal and expansion of chemotherapy-resistant BCSCs

**Figure 7:**
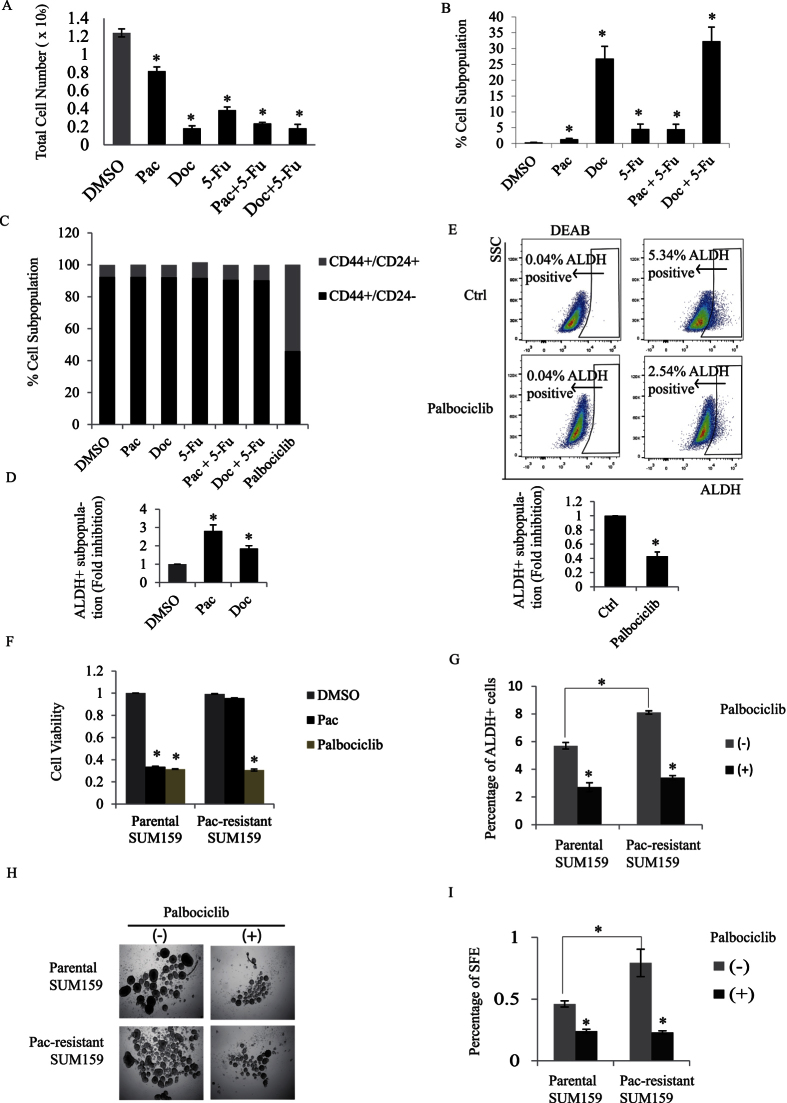
(**A**) SUM159 cells were treated with DMSO (vehicle) and several chemotherapeutic agents including 10 nM paclitaxel (Pac), 10 nM docetaxel (Doc), and 10 nM 5-fluorouracil (5-Fu) alone or in combination for 4 days. Cell viability following drug treatments was assessed by measuring cell numbers. (**B**) SUM159 cells were labelled with PKH26 and treated with the different chemotherapy drug treatments for 4 days. Flow analysis of PKH26 positive cells were performed and percentage of PKH26+ cells was quantified using flow Jo. (**C**) SUM159 cells were treated with the different drugs and percentages of the different CD44^+/−^/CD24^+/−^ subpopulations were quantified and graphed. (**D**) SUM159 cells were treated with DMSO, Pac, or Doc for 4 days. ALDEFluor assay was performed and percentage of ALDH+ cells was analyzed by flow cytometry. (**E**) SUM159 cells were cultured in the presence or the absence of palbociclib and the percentage of ALDH+ cells was assessed and quantified using flow cytometry. The ALDH inhibitor DEAB was used as a negative control for gating purposes. (**F**) SUM159 cells were treated with several round of 10 nM Paclitaxel until repopulated SUM159 cells became completely insensitive to fresh paclitaxel. Parental SUM159 and Paclitaxel-resistant SUM159 cells were then treated with DMSO, Paclitaxel, or Palbociclib for 2 days. Growth inhibition was then assessed by measuring cell numbers. (**G**) Parental SUM159 and Paclitaxel-resistant SUM159 cells were treated with or without palbociclib for 2 days. Percentage of ALDH+ cells were assessed and quantified. (**H**,**I**) Parental SUM159 and Paclitaxel-resistant SUM159 cells were treated or not with palbociclib and tumorsphere formation efficiency was assessed and quantified.

TNBC patients are often resistant to chemotherapy and suffer early relapse within 5 years of diagnosis. To examine whether drug-resistance and tumor relapse associate with an increase population of BCSCs, we first assessed the effect of several chemotherapeutic agents including paclitaxel (Pac), docetaxel (Doc), and 5-fluorouracil (5-Fu) on BCSC populations in TNBC cells. Although these drug treatments with Pac, Doc, or 5-Fu individually or in combination decreased total cell number of SUM159 cells (Fig. 7A), they in fact increased the percentage of slow-dividing PKH26+ BCSCs that have potent tumor-initiating capacity (Fig. 7B)^23^. Moreover, unlike the CDK4 inhibitor palbociclib, the chemotherapy drugs failed to eliminate or reduce the CD44^+^/CD24^−^ BCSC population (Fig. 7C). As mentioned earlier, another sub-population of cancer stem cells has been characterized in breast tumors (ALDH^+^ cells). The ALDH^+^ BCSCs have been reported to be enriched following chemotherapy treatment with paclitaxel in TNBC patients^33^. We thus compared the effect of the chemotherapy drugs Pac and Doc on the ALDH^+^ population and found that treatment with both chemotherapy drugs, not only did not reduce the ALDH+ population but in fact led to a clear increase in the number of ALDH^+^ cells (Fig. 7D). On the contrary, when cells were treated with the CDK4 inhibitor, palbociclib, we found a significant decrease in the number of ALDH^+^ cells (Fig. 7E). These results indicate that although chemotherapy treatments effectively eliminate the majority of the bulk tumor cells but they have no effect on targeting CD44^+^/CD24^−^ BCSCs. Moreover, chemotherapy drugs rather expand the cancer stem cell population by specifically increasing the ALDH^+^ BCSC population. On the contrary, the CDK4 kinase inhibitor potently eliminated or reduced the number of both CD44^+^/CD24^−^ and ALDH^+^ BCSC populations.

Together, these results indicate that not only CDK4 is highly expressed in BCSC-enriched TNBCs, but that its kinase activity is required for the maintenance of CD44^+^/CD24^−^ and ALDH^+^ BCSC subpopulations. Considering that these two BCSC populations exhibit strong local tumor- and distant metastasis-initiating capacities^5^, our results, thus contribute to our understanding of why chemotherapy treatments often lead to drug resistance and subsequent tumor recurrence and metastasis. They also suggest that pharmacological CDK4 kinase inhibitors (i.e. palbociclib) may prove worthy for the development of novel therapies aiming at efficiently targeting and eliminating cancer stem cells in TNBCs.

Having shown that CDK4 regulates BCSCs expansion and promotes BCSC self-renewal capacity, we next investigated the potential role of CDK4 in BCSC chemotherapy resistance, with a long term goal of identifying potential new clinical therapeutic approaches for tumor relapse in TNBC patients. To address this, we generated paclitaxel-resistant SUM159 cells by treating cells with several cycles of paclitaxel. As shown in Fig. 7F, as measured by growth inhibition assay, SUM159 cells became completely resistant to paclitaxel after three rounds of treatments and recovery. We then examined whether the drug palbociclib could be a potential treatment for chemotherapy-resistant TNBCs. Interestingly, the Pac-resistant cells were highly responsive to the CDK4 inhibitor palbociclib (Fig. 7F). Moreover, while paclitaxel-resistant cells displayed an increased ALDH^+^ cell number, the percentage of these BCSC ALDH^+^ cells was strongly reduced in response to palbociclib treatment (Fig. 7G). To finally assess the effect of inhibiting CDK4 kinase activity on self-renewal capacity of chemotherapy-resistant cells, we measured the tumorsphere formation efficiency. As shown in Fig. 7H,I, paclitaxel-resistant cells have a higher self-renewal potential as reflected by the increased SFE compared to parental cells. However, palbociclib treatment of the cells potently inhibited their self-renewal potential as indicated by the reduced SFE numbers.

These results highlight CDK4 as a critical mediator of TNBC resistance to chemotherapy and further suggest the use of the CDK4 inhibitor, palbociclib as a potential drug for not only eliminate BCSCs but also further prevent tumor recurrence and chemotherapy resistance in human TNBCs.

## Discussion

Current therapeutic treatments have significantly reduced the mortality in breast cancer patients^34^. The improvement of patient survival outcome is largely due to the development of targeted therapy for specific subtypes of breast tumors, such as aromatase inhibitors and hormonal agents for hormone sensitive tumors, as well as Her2 targeting antibody trastuzumab for HER2 overexpression tumors. TNBCs with high histologic grade and poor survival outcome still have no molecular-targeted therapies and very limited success with the use of conventional chemotherapies, such as paclitaxel. Despite adjuvant chemotherapy, less than 30% of TNBC patients survive within 5 years of diagnosis due to local and distant tumor recurrence^22^. Thus, identification of novel molecular targets is still critical for the development of successful therapies for this very aggressive and deadly subtype of breast cancer.

The CDK4 gene is frequently amplified in human cancer and its deregulation has been observed in many types of human cancers. However, the relationship between CDK4 and clinical prognosis for breast cancer remains unclear. In this study, we analyzed the association of CDK4 gene expression with the prognostic features and survival outcomes of breast tumors. We show that CDK4 expression is elevated in the most aggressive TNBC subtypes and strongly associated with the basal-like subtype, ER negative and higher grade of primary breast tumors, all of which represent poor prognostic features of TNBCs. Indeed, high expression of CDK4 predicted both poor overall survival and relapse-free survival outcomes in breast cancer patients. Together, our findings support that the notion that CDK4 can serve as an important prognostic marker and survival outcome indicator for TNBC patients.

CSCs are thought to play critical roles in tumor recurrence, metastasis, and drug resistance. These subpopulations of cancer cells have emerged as potential cellular targets for clinical therapeutic strategies. BCSCs are largely enriched in basal-like, ER- and high grade tumors, and in line with these observations, we found a clear correlation between CDK4 gene expression and these histological features. We also describe a new function for CDK4 in maintaining BCSC self-renewal. Our data further indicate that high level of CDK4 expression may contribute to poor prognosis features and poor clinical outcomes by increasing the cancer stem cell numbers. Indeed, knocking down CDK4 gene expression selectively reduced the CD44^+^/CD24^−^ and ALDH^+^ BCSC subpopulations of TNBC cells and inhibited their self-renewal capacity. Several CDK inhibitors are currently undergoing clinical trials for many types of cancer including breast cancer, glioblastoma, leukaemia, pancreatic carcinoma^35^. These clinical studies showed that inhibition of CDK4/6 kinase activity prevents proliferation and tumor growth. Early 2015, Pfizer CDK4/6 inhibitor, palbociclib received accelerated FDA approval for the treatment of ER^+^/HER-metastatic breast cancer, in combination with the aromatase inhibitor letrozole. The phase II studies in ER+/HER- patients revealed a significant improved outcome for these patients, with a median progression free survival rate improving from 10 months for letrozole alone to 20 months for the combinatorial treatment^25^. In the present study, we found that blocking CDK4 kinase activity using palbociclib reduced BCSC cell numbers and their self-renewal capacity in TNBCs. These results suggest a central role for CDK4 in BCSC generation and in the regulation of TNBC expansion. They also highlight the potential beneficial use of palbociclib in combinatorial therapies for efficiently treating TNBC patients.

Initial studies using palbociclib in hormone responsive breast tumor patients revealed an expected beneficial, yet unexplained, effect of the drug. Indeed, even though blocking cell cycle should only block tumor from further growing, treatment with the CDK4 inhibitor palbociclib revealed that it could also shrink tumors^25^. Our data clearly indicate that blocking CDK4 kinase activity leads to inhibition of BCSC self-renewal and significantly reduces cancer stem cell numbers. Thus, palbociclib-driven elimination of the BCSC subpopulations in these tumors will not only induce tumor stasis but also likely contribute to tumor regression.

A recent study in TNBC showed that the number of cancer stem cells increased after treatment with the chemotherapeutic drug paclitaxel^33^. Because CSCs possess self-renewal and tumor initiation capacity as well as a drug-resistant nature, the high incidence of tumor relapse in these TNBC patients is likely due to the increase in the CSC population. Comparison of the gene expression profiles of CD44^+^ and CD24^+^ cells revealed that CD44^+^ cells specifically expressed multiple stem cell markers, which expression correlate with poor survival outcomes in breast cancer patients^36^. Furthermore, the proportion of CD44^+^/CD24^−/low^ tumor cells in breast cancer patients was found to increase after chemotherapy treatment^37^, suggesting these CSCs are resistant to chemotherapy. In the present study, we analyzed the effects of various chemotherapy drugs, alone or in combination and found that while sufficient enough to eliminate the bulk tumor cells, they actually failed to decrease BCSC numbers and in fact contributed to an enrichment of the ALDH^+^ BCSC cell population numbers as well as to an increase of their self-renewal capacity, further contributing to tumor relapse. Moreover, long-term treatment of TNBC cells with paclitaxel resulted in their complete resistance to the chemotherapy drug. Interestingly, we were able to efficiently eliminate these paclitaxel-resistant TNBC cells and reduce their self-renewal capacity using the CDK4 inhibitor palbociclib.

Together, these results highlight CDK4 as a potent self-renewal regulator of BCSCs and as a critical mediator of TNBC resistance to chemotherapy. Our study also highlights the promising use of CDK4 pharmacological inhibitors as a potential new drugs to eliminate cancer stem cells and further prevent tumor recurrence in human TNBCs.

## Methods

### Cell culture

Human breast cancer cell line SUM159PT was cultured in F-12 HAM’S nutrient mixture (HyClone Laboratories, Inc. Logan, Utah, USA) containing 5% FBS, 5 μg/ml insulin (Sigma-Aldrich, St. Louis, MO, USA), and 1 μg/ml hydrocortisone (Sigma). Human breast cancer cell line MDA and SCP2 were cultured in DMEM containing 10% fetal bovine serum (FBS) and 2 mM L-glutamine. All cell lines were grown at 37 °C in 5% CO_2_.

### Lentiviral infection

HEK293T cells were transfected with scrambled shRNA, cyclin D1 shRNA, CDK4 shRNA, CDK6 shRNA and packaging plasmids (psPAX2 and pMD2.G). After 36 hours posttransfection, cell supernatants containing shRNA lentiviruses were collected. SUM159, MDA, SCP2 cells were infected with lentiviral shRNAs in the presence of 8 μg/ml polybrene.

### Tumorsphere formation assay

Monolayer cells or tumorspheres were enzymatically dissociated into single cells with 0.05% trypsin-EDTA. Cells were plated at 10,000 cells per well in a 24-well low-attachment plate (Corning). Cells were grown for 7 days in DMEM/F12 supplemented with B27 (Invitrogen) in the presence of 10 ng/ml EGF and 10 ng/ml bFGF. Where indicated, the CDK4 inhibitor palbocilib (Sigma) was added at a final concentration of 100 nM. Tumorsphere-forming efficiency was calculated as the number of spheres divided by the number of singles cells seeded, expressed as a percentage.

### Flow cytometry analysis

Adherent cells were dissociated into single cells by trypsin-EDTA and filtered through a 40 μm nylon mesh (BD Biosciences, San Diego, CA). 500,000 cells were washed with PBS containing 0.5% BSA (FACS buffer), incubated with anti-CD44 conjugated to APC, anti-CD24 conjugated to PE, anti-MUC1 conjugated to APC for 30 minutes on ice. All antibodies were from BD Biosciences. Isotype-matched conjugated non-immune antibodies were used as negative controls. Cells were then washed with FACS buffer for 3 times. After washing, cells were analyzed with Accuri C6 flow cytometer (BD Biosciences) and Flowjo software (Tree Star Inc.). Single CD44+/CD24− or CD44+/CD24+ cell was sorted using FACSAria into a 96-well low attachment plate containing DMEM/F12 supplemented with B27 (Invitrogen) in the presence of 20 ng/ml EGF and 20 ng/ml bFGF. Cells were then cultured for 10 days and tumorspheres were counted.

ALDEFLUOR assay was performed as described in the manufacturer’s protocol, 1 × 10^6^ SUM159 cells were centrifuged and resuspended in 1 ml ALDH assay buffer. 5 μl substrate was added into the cell suspension. For negative control, 500 μl cell suspension were then transferred into a new tube containing 5 μl DEAB. Cells were then incubated for 40 min at 37 °C. Percentage of ALDH+ cells was analyzed with Accuri C6 flow cytometer and Flowjo software.

PKH 26 labelling was performed according to the manufacturer’s protocol (Sigma). Briefly, 2 × 10^7^ SUM159 cells were suspended in 1 ml diluent C. 4 μl PKH26 reagent was added into a new tube containing 1 ml diluent C and mixed with cell suspension for 5 min. The cell suspension were then washed with 10% FBS medium and plated for cell culture. Cells were then treated with DMSO and chemotherapy drug 10 nM paclitaxel, 10 nM docetaxel, and 10 nM 5-fluorouracil for 2 or 4 days. For the generation of paclitaxel-resistant cells, cells were treated with paclitaxel for every two days and replaced with complete medium for recovery for two days.

### Western blot analysis

Cells derived from monolayer or mammosphere were extracted with lysate buffer containing10 mm Tris-HCl, pH 7.5, 5 mm EDTA, 150 mm NaCl, 30 mm sodium pyrophosphate, 50 mm sodium fluoride, 1 mm sodium orthovanadate, 1% Triton X-100 and protease inhibitors (1 mm phenylmethylsulfonyl fluoride, 10 μg/ml leupeptin hydrochloride, 10 μg/ml aprotinin and 10 μg/ml pepstatin A) at 4 °C. After cell lysates were centrifuged at 14,000 rpm for 15 minutes at 4 °C, the concentration of total protein was quantified using a BCA protein assay kit (Thermo Scientific, Rockford, IL, USA). Cell lysates were boiled with 6× sodium dodecyl sulfate (SDS) Laemmli sample buffer for five minutes and subjected to immunoblot analysis using antibodies against cyclin D1 (Neomarker), phospho-retinoblastoma Ser780 (Cell Signaling Technology), and anti-CDK4 (Santa Cruz Biotechnology).

### Real-time PCR

SUM159 and MDA cells were lysate in TRIzol reagents (Invitrogen) and total RNA was extracted according to manufacturer’s protocol. Reverse transcription was performed using M-MLV reverse transcriptase (Invitrogen) and random primers as per the manufacturer’s instructions. Real-time PCRs were carried out using SsoFast^TM^ EvaGreen^®^ Supermix (Bio-Rad) in a Rotor Gene 6000 PCR detection system (MBI Lab Equipment). PCR conditions for amplifying nanog, pou5f1, sox2, KRT8, CDH1 and ACTA2 gene were as follows: 95 °C for 30 s, 40 cycles (95 °C for 5 s and 60 °C for 20 s).

### Immunofluorescence microscopy

SUM159 cells were grown on coverslips, fixed with 3.7% formaldehyde for 10 minutes and permeabilized in 0.1% Triton X-100 for 3 minutes. Cell were then washed with PBS and blocked for 1 hr in 2% BSA. Cells were incubated with an anti-E-cadherin specific antibody for one hour, washed with PBS and incubated with Alexa Fluor^®^488 goat anti-rabbit antibody for one hour. Stained coverslips were mounted with SlowFade^®^ Gold antifade reagent with DAPI. Confocal analysis was performed using a Zeiss LSM 510 Meta Axiovert confocal microscope using 63× objective.

### BMP4 promoter luciferase assay

SUM159 cells were infected with scrambled or CDK4 shRNA. After two days infection, cells were then transfected with a reporter construct containing 3.36 kb of the 5′ BMP4 gene promoter regulatory region fused to the luciferase gene (3.36 kb-BMP4-luc) was provided by Dr. Daniel Chung [39]. Luciferase activity of the promoter construct was assessed using a luminometer.

### Caspase Glo 3/7 Assay

Cells were plated in 96-well plates at 5000 cells/100 μL in medium supplemented with 2% FBS. Caspase 3/7 activity was measured using the Caspase Glo 3/7 Assay (Promega) according to manufacturer’s instructions. Cells were incubated with equal volumes of medium and Caspase Glo reagent for 30 minutes at room temperature, and luminescence was measured using the EG & G Berthold luminometer (Bad Wildbad, Germany).

### Statistical analyses

All results are presented as the mean ± SEM for at least three repeated individual experiments. The difference between groups was analyzed using Student’s *t*-test, and *P < 0.05 was considered statistically significant.

## Additional Information

**How to cite this article**: Dai, M. *et al.* CDK4 regulates cancer stemness and is a novel therapeutic target for triple-negative breast cancer. *Sci. Rep.* **6**, 35383; doi: 10.1038/srep35383 (2016).

## Supplementary Material

Supplementary Information

## References

[b1] Magee J. A., Piskounova E. & Morrison S. J. Cancer stem cells: impact, heterogeneity, and uncertainty. Cancer cell 21, 283–296, doi: 10.1016/j.ccr.2012.03.003 (2012). 22439924 PMC4504432

[b2] Visvader J. E. & Lindeman G. J. Cancer stem cells: current status and evolving complexities. Cell stem cell 10, 717–728, doi: 10.1016/j.stem.2012.05.007 (2012). 22704512

[b3] Reya T., Morrison S. J., Clarke M. F. & Weissman I. L. Stem cells, cancer, and cancer stem cells. Nature 414, 105–111, doi: 10.1038/35102167 (2001). 11689955

[b4] Dean M., Fojo T. & Bates S. Tumour stem cells and drug resistance. Nature reviews. Cancer 5, 275–284, doi: 10.1038/nrc1590 (2005). 15803154

[b5] Al-Hajj M., Wicha M. S., Benito-Hernandez A., Morrison S. J. & Clarke M. F. Prospective identification of tumorigenic breast cancer cells. Proceedings of the National Academy of Sciences of the United States of America 100, 3983–3988, doi: 10.1073/pnas.0530291100 (2003). 12629218 PMC153034

[b6] Singh S. K. *et al.* Identification of a cancer stem cell in human brain tumors. Cancer research 63, 5821–5828 (2003). 14522905

[b7] Cammareri P. *et al.* Isolation and culture of colon cancer stem cells. *Methods*in cell biology 86, 311–324, doi: 10.1016/S0091-679X(08)00014-9 (2008). 18442654

[b8] Hurt E. M., Kawasaki B. T., Klarmann G. J., Thomas S. B. & Farrar W. L. CD44+ CD24(-) prostate cells are early cancer progenitor/stem cells that provide a model for patients with poor prognosis. British journal of cancer 98, 756–765, doi: 10.1038/sj.bjc.6604242 (2008). 18268494 PMC2259168

[b9] Balic M. *et al.* Most early disseminated cancer cells detected in bone marrow of breast cancer patients have a putative breast cancer stem cell phenotype. Clinical cancer research: an official journal of the American Association for Cancer Research 12, 5615–5621, doi: 10.1158/1078-0432.CCR-06-0169 (2006). 17020963

[b10] Ginestier C. *et al.* ALDH1 is a marker of normal and malignant human mammary stem cells and a predictor of poor clinical outcome. Cell stem cell 1, 555–567, doi: 10.1016/j.stem.2007.08.014 (2007). 18371393 PMC2423808

[b11] Massague J. G1 cell-cycle control and cancer. Nature 432, 298–306, doi: 10.1038/nature03094 (2004). 15549091

[b12] Dai M. *et al.* A novel function for p21Cip1 and acetyltransferase p/CAF as critical transcriptional regulators of TGFbeta-mediated breast cancer cell migration and invasion. Breast cancer research: BCR 14, R127, doi: 10.1186/bcr3322 (2012). 22995475 PMC4053104

[b13] Kozar K. *et al.* Mouse development and cell proliferation in the absence of D-cyclins. Cell 118, 477–491, doi: 10.1016/j.cell.2004.07.025 (2004). 15315760

[b14] Beroukhim R. *et al.* The landscape of somatic copy-number alteration across human cancers. Nature 463, 899–905, doi: 10.1038/nature08822 (2010). 20164920 PMC2826709

[b15] Lange C. & Calegari F. Cdks and cyclins link G1 length and differentiation of embryonic, neural and hematopoietic stem cells. Cell Cycle 9, 1893–1900 (2010). 20436288 10.4161/cc.9.10.11598

[b16] Jeselsohn R. *et al.* Cyclin D1 kinase activity is required for the self-renewal of mammary stem and progenitor cells that are targets of MMTV-ErbB2 tumorigenesis. Cancer cell 17, 65–76, doi: 10.1016/j.ccr.2009.11.024 (2010). 20129248 PMC2818730

[b17] Artegiani B., Lindemann D. & Calegari F. Overexpression of cdk4 and cyclinD1 triggers greater expansion of neural stem cells in the adult mouse brain. The Journal of experimental medicine 208, 937–948, doi: 10.1084/jem.20102167 (2011). 21482697 PMC3092341

[b18] Lange C., Huttner W. B. & Calegari F. Cdk4/cyclinD1 overexpression in neural stem cells shortens G1, delays neurogenesis, and promotes the generation and expansion of basal progenitors. Cell stem cell 5, 320–331, doi: 10.1016/j.stem.2009.05.026 (2009). 19733543

[b19] Hu Z. *et al.* The molecular portraits of breast tumors are conserved across microarray platforms. BMC genomics 7, 96, doi: 10.1186/1471-2164-7-96 (2006). 16643655 PMC1468408

[b20] Parker J. S. *et al.* Supervised risk predictor of breast cancer based on intrinsic subtypes. Journal of clinical oncology: official journal of the American Society of Clinical Oncology 27, 1160–1167, doi: 10.1200/JCO.2008.18.1370 (2009). 19204204 PMC2667820

[b21] Dai M. *et al.* Cyclin D1 cooperates with p21 to regulate TGFbeta-mediated breast cancer cell migration and tumor local invasion. Breast cancer research: BCR 15, R49, doi: 10.1186/bcr3441 (2013). 23786849 PMC4053239

[b22] Dent R. *et al.* Triple-negative breast cancer: clinical features and patterns of recurrence. Clinical cancer research: an official journal of the American Association for Cancer Research 13, 4429–4434, doi: 10.1158/1078-0432.CCR-06-3045 (2007). 17671126

[b23] Pece S. *et al.* Biological and molecular heterogeneity of breast cancers correlates with their cancer stem cell content. Cell 140, 62–73, doi: 10.1016/j.cell.2009.12.007 (2010). 20074520

[b24] Gyorffy B. *et al.* An online survival analysis tool to rapidly assess the effect of 22,277 genes on breast cancer prognosis using microarray data of 1,809 patients. Breast Cancer Res Treat 123, 725–731, doi: 10.1007/s10549-009-0674-9 (2010). 20020197

[b25] Turner N. C. *et al.* Palbociclib in Hormone-Receptor-Positive Advanced Breast Cancer. The New England journal of medicine, doi: 10.1056/NEJMoa1505270 (2015). 26488700

[b26] Geng Y. *et al.* Deletion of the p27Kip1 gene restores normal development in cyclin D1-deficient mice. Proceedings of the National Academy of Sciences of the United States of America 98, 194–199, doi: 10.1073/pnas.011522998 (2001). 11134518 PMC14567

[b27] Todaro M. *et al.* CD44v6 is a marker of constitutive and reprogrammed cancer stem cells driving colon cancer metastasis. Cell stem cell 14, 342–356, doi: 10.1016/j.stem.2014.01.009 (2014). 24607406

[b28] Charafe-Jauffret E. *et al.* Breast cancer cell lines contain functional cancer stem cells with metastatic capacity and a distinct molecular signature. Cancer research 69, 1302–1313, doi: 10.1158/0008-5472.CAN-08-2741 (2009). 19190339 PMC2819227

[b29] Fillmore C. M. & Kuperwasser C. Human breast cancer cell lines contain stem-like cells that self-renew, give rise to phenotypically diverse progeny and survive chemotherapy. Breast cancer research: BCR 10, R25, doi: 10.1186/bcr1982 (2008). 18366788 PMC2397524

[b30] Ponti D. *et al.* Isolation and *in vitro* propagation of tumorigenic breast cancer cells with stem/progenitor cell properties. Cancer research 65, 5506–5511, doi: 10.1158/0008-5472.CAN-05-0626 (2005). 15994920

[b31] Hinohara K. *et al.* ErbB receptor tyrosine kinase/NF-kappaB signaling controls mammosphere formation in human breast cancer. Proceedings of the National Academy of Sciences of the United States of America 109, 6584–6589, doi: 10.1073/pnas.1113271109 (2012). 22492965 PMC3340023

[b32] Zhang L. *et al.* BMP4 administration induces differentiation of CD133+ hepatic cancer stem cells, blocking their contributions to hepatocellular carcinoma. Cancer research 72, 4276–4285, doi: 10.1158/0008-5472.CAN-12-1013 (2012). 22773665

[b33] Bhola N. E. *et al.* TGF-beta inhibition enhances chemotherapy action against triple-negative breast cancer. The Journal of clinical investigation 123, 1348–1358, doi: 10.1172/JCI65416 (2013). 23391723 PMC3582135

[b34] Berry D. A. *et al.* Effect of screening and adjuvant therapy on mortality from breast cancer. N Engl J Med 353, 1784–1792, doi: 10.1056/NEJMoa050518 (2005). 16251534

[b35] Musgrove E. A., Caldon C. E., Barraclough J., Stone A. & Sutherland R. L. Cyclin D as a therapeutic target in cancer. Nature reviews. Cancer 11, 558–572, doi: 10.1038/nrc3090 (2011). 21734724

[b36] Shipitsin M. *et al.* Molecular definition of breast tumor heterogeneity. Cancer cell 11, 259–273, doi: 10.1016/j.ccr.2007.01.013 (2007). 17349583

[b37] Li X. *et al.* Intrinsic resistance of tumorigenic breast cancer cells to chemotherapy. Journal of the National Cancer Institute 100, 672–679, doi: 10.1093/jnci/djn123 (2008). 18445819

