# Supplementary material for: CDK4 regulates cancer stemness and is a novel therapeutic target for triple-negative breast cancer
Source: Sci Rep. 2016 Oct 19;6:35383. doi: 10.1038/srep35383 (PMC5069501; doi:10.1038/srep35383)
Supplement: Supplementary Information [file srep35383-s1.pdf]

CDK4 regulates cancer stemness and is a novel therapeutic target for triple-negative breast cancer

Meiou Dai[1], Chenjing Zhang[1], Ayad Ali[1], Xinyuan Hong[1], Tian Jun[1], Chieh Lo[1], Nadège Fils-Aimé[1], Sergio A. Burgos[2], Suhad Ali[1], Jean Jacques Lebrun[1]*

Supplementary Figure Legends

Figure S1: A, MDA and SCP2 cells were treated with or without 100 nM of palbociclib for 2 days and subjected to light microscopy (10x objective). **B**, Gene expressions of cytokeratin 8 (KRT8), E-cadherin (CDH1), and alpha smooth muscle actin (ACTA2) in MDA cells were measured using RT-PCR. **C**, MDA cells were infected with scrambled, cyclin D1, CDK4 or CDK6 shRNA overexpressing lentiviruses and imaged by light microscopy.

Figure S2: A, SUM159 cells were infected with scrambled and CDK6 shRNA. Percentage of SFE was measured and graphed. **B**, Gene expression of CDK6 was measured by RT-PCR in infected SUM159 cells. **C**, Gene set analysis of CDK6 in normal tissues and primary tumors including basal-like, HER2+, luminal A, luminal B subtype tumors. **D**, CDK6 expression was assessed by Kaplan-Meier survival analysis for 5-year overall survival and relapse free survival outcome of breast cancer patients and basal-like patients.

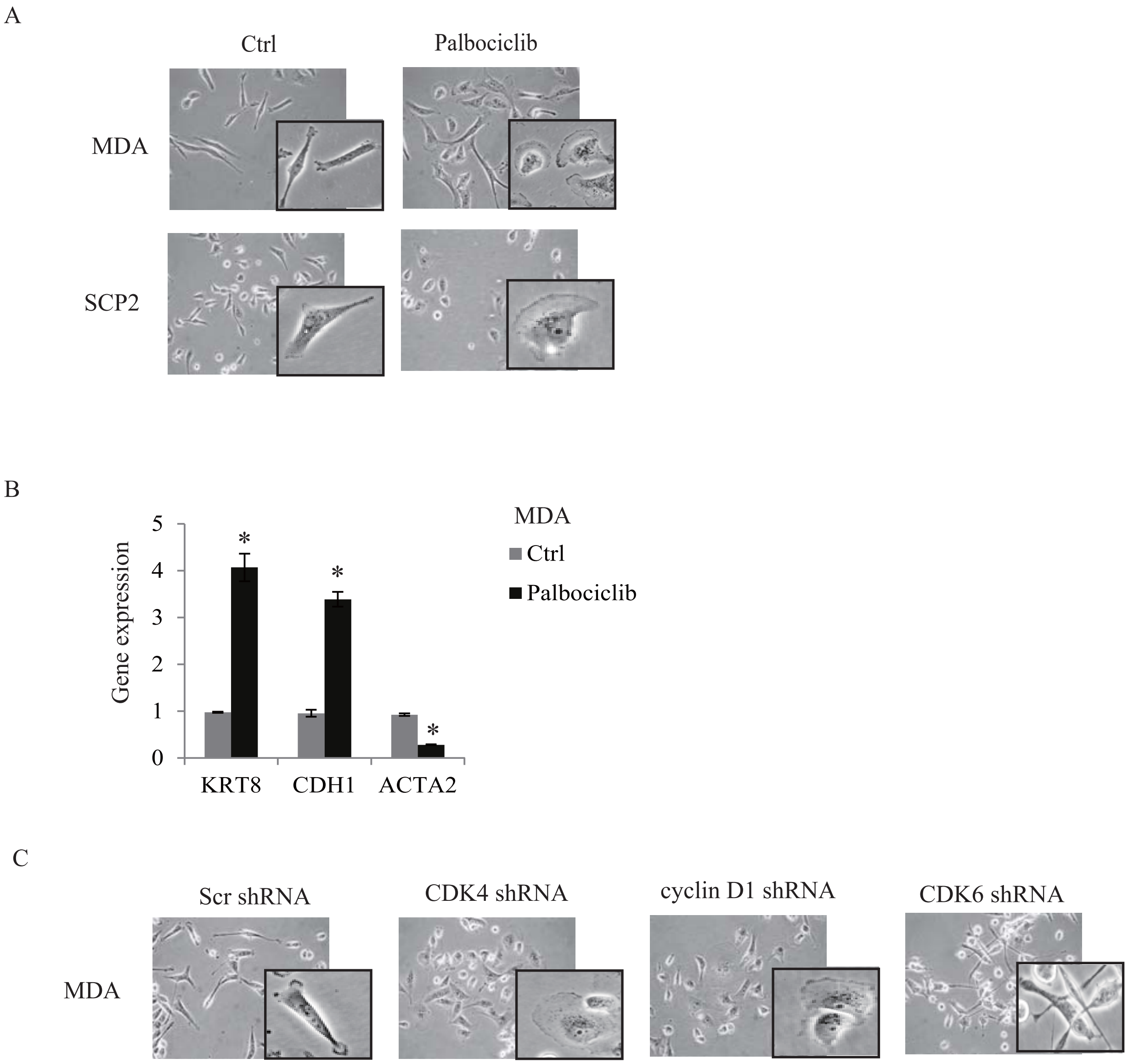
A
Ctrl
Palbociclib
MDA
SCP2
B
Gene expression
5
4
3
2
1
0
*
*
*
KRT8
CDH1
ACTA2
MDA
Ctrl
Palbociclib
C
Scr shRNA
CDK4 shRNA
cyclin D1 shRNA
CDK6 shRNA
MDA

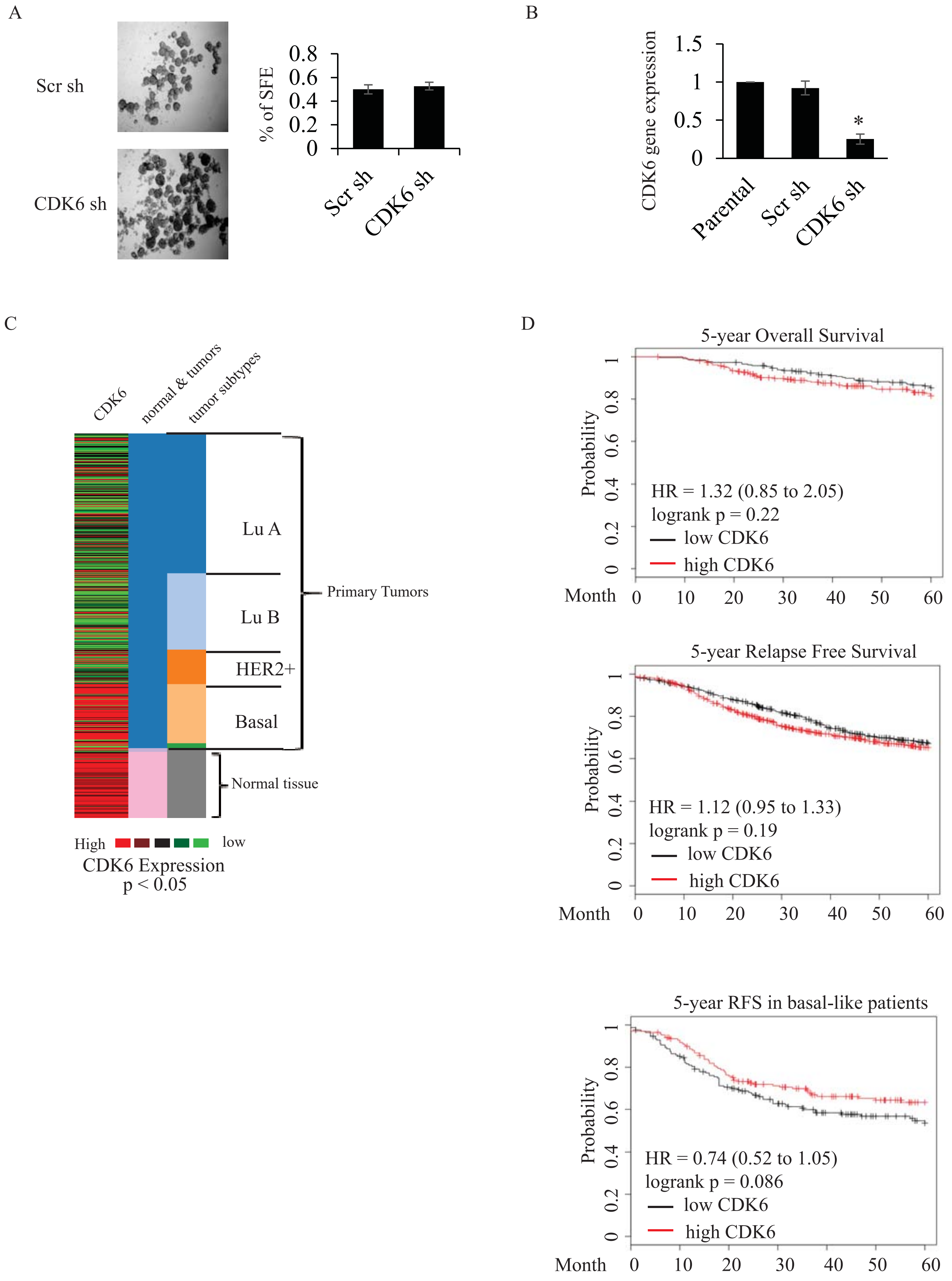

A
Scr sh
CDK6 sh
% of SFE
0.8
0.6
0.4
0.2
0
Scr sh
CDK6 sh
B
CDK6 gene expression
1.5
1
0.5
0
*
Parental
Scr sh
CDK6 sh
C
CDK6
normal & tumors
tumor subtypes
Lu A
Lu B
HER2+
Basal
Primary Tumors
Normal tissue
High
low
CDK6 Expression
p < 0.05
D
5-year Overall Survival
Probability
HR = 1.32 (0.85 to 2.05)
logrank p = 0.22
low CDK6
high CDK6
Month
0 10 20 30 40 50 60
5-year Relapse Free Survival
Probability
HR = 1.12 (0.95 to 1.33)
logrank p = 0.19
low CDK6
high CDK6
Month
0 10 20 30 40 50 60
5-year RFS in basal-like patients
Probability
HR = 0.74 (0.52 to 1.05)
logrank p = 0.086
low CDK6
high CDK6
Month
0 10 20 30 40 50 60
